# Are you in top 1% (1‰)?

**DOI:** 10.1007/s11192-017-2526-4

**Published:** 2017-09-23

**Authors:** Marek Kosmulski

**Affiliations:** Lublin University of Technology, Lublin, Poland

**Keywords:** Citation, Assessment of authors, Assessment of publications, Scientific degree

## Abstract

A new simple bibliometric indicator is based on the number of highly cited papers (as defined by WoS^®^). It can be used to assess individuals, journals and universities. Unlike most other citation-based-indicators it equalizes the chances of young scientists (vs. their more experienced colleagues) and of scientists working in less-popular disciplines. The ranking of scientists based on the new indicator is not correlated with the rankings based upon the number of citations or on the Hirsch-index.

**Electronic supplementary material:**

The online version of this article (doi:10.1007/s11192-017-2526-4) contains supplementary material, which is available to authorized users.

## Introduction

Since the seminal work of Garfield (1955) the scientific output is assessed by its impact (the number of citations) rather than by the quantity (number of publications). The impact is used in assessment of individuals, institutions and scientific journals, and the journal impact factor is still the most common indicator of the quality of scientific journals (Garfield 2006).

On top of the total number of citations, many other citation-based indicators have been considered (Bornmann et al. 2011; Schreiber 2010). The possibility of having received many citations by production of numerous low-impact papers is among the most-frequently criticized properties of the total number of citations as the method of assessment of the scientific output of individuals or of institutions. Therefore the total number of citations is often replaced by the number of “successful papers” (or by a number of citations thereof) which can be defined in different ways. The Hirsch (2005)-index defining the successful paper as a paper whose number of citations is ≥ its rank by # of citations is the most well-known definition of successful paper. The other common definitions of successful papers have been discussed in detail elsewhere (Kosmulski 2011). In spite of different definitions of successful papers, most citation-based indicators are highly correlated (Bornmann et al. 2011; Schreiber 2010).

In June 2017 WoS^®^ introduced a new functionality, which is a one-click extraction of highly cited papers (papers published over the recent decade, which received enough citations as of certain date, which is about 3 months before the date of the search, to place them in the top 1% in certain field for certain publication year) and of hot papers (papers published in the past 2 years which received enough citations as of certain date, which is about 3 months before the date of the search, to place them in the top 0.1% of papers in its field) from any set of papers. Most hot papers are also highly cited papers, but only a few highly cited papers are also hot papers. The highly cited papers and hot papers have been marked as such also in older versions of the users’ interface of WoS^®^, but till June 2017 they could only be extracted manually, and this extraction was tedious in large sets of data. Nevertheless, numerous studies were devoted to different aspects of highly cited papers.

Noorhidawati et al. (2017) observed a linear increase with publication year of the number of highly cited papers with Malayan affiliation. 52% of these papers represented engineering and technology, and only 16% represented medicine. Most papers had two to five authors, and the fraction of papers authored by 10 or more co-authors was only 25%.

Docampo and Cram (2017) studied the number of highly cited papers from Canada, Australia, Italy, and Spain published in 2014–2015. In spite of similar total numbers of papers from the 4 countries, Canada had twice as many highly cited papers as each of three other countries. Miryairi and Chang (2012) analyzed the ratio between highly cited papers and total published papers in multiple countries over the periods 2000–2004 and 2005–2009. Switzerland was the top country with 2.2% of highly cited papers followed by USA and Denmark (1.8% each), and Poland had only 0.6% of highly cited papers (less than the expected 1%). In most countries the fraction of highly cited papers was rather stable in time, but a few countries showed a remarkable increase, e.g., from 0.7 in 2000–2004 to over 1% in 2005–2009 in case of Singapore.

Pislyakov and Shukshina (2014) used highly cited papers to identify the top scientific institutions in Russia. They also analyzed Russian highly cited papers published in 2000–2009 by discipline. Over 50% of these papers were in physics, and the contributions of other disciplines including engineering, clinical medicine, and chemistry were less than 10% each. The above examples indicate that highly cited papers were mainly used to assess the performance at country level or at institution level, but not at the level of individuals. This should be emphasized that the term “highly cited paper*” is often used in the literature in a meaning different from the above definition by WoS^®^ (e.g., Plomp 1990).

The new functionality inspired the present author to propose a new bibliometric indicator, based on the # of highly cited papers and of hot papers produced by certain individual, institution, or published in certain scientific journal.

Let us define a successful paper as a highly cited paper according to the above definition by WoS^®^, and let us assess scientists by a # of highly cited papers. Due to the aforementioned one-click functionality, recently introduced by WoS^®^ such an assessment can be completed in a short time. The possible advantage of the new method of assessment is equalization of the chances of young scientists (papers older than 10 years are not taken into account, anyway) and of scientists working in less-popular disciplines (their papers need fewer citations to be highly cited). The properties of the new indicator are studied using a set of papers from one city.

## Methods

The WoS^®^ core collection was accessed on July 3, and on July 17, 2017. The results were stored, and they were processed afterwards. We are interested in papers with “Lublin” in their address field. Lublin is a city in Poland with 5 large universities, one large research institute, and several medical facilities, which also perform research work. The present author lives in that city and knows many top-scientists from that city personally.

The scientists who have published at least one highly cited paper or at least one hot paper are characterized by their scientific degree. In principle there are four categories of degrees in Poland, which were assigned the following values: 3 (professor), 2 (Dr.Sci.), 1 (Ph.D.), and 0 (M.Sci.). Foreign citizens who worked in Lublin as post-docs, and authored papers under Polish affiliation are counted as Ph.D.

The scientists who have published at least one highly cited paper or at least one hot paper are characterized by their total number of citations and their *h*-index. Those were taken from Scopus. The advantage of Scopus versus WoS^®^ is in easier extraction of certain individual from a set of homonymous authors.

The scientists who have published at least one highly cited paper or at least one hot paper are characterized by their institution, which is one of the top universities in Lublin, marked *u*1, *u*2, *u*3, *u*4 and *u*5, a research institute marked as *i*1, and one of the medical facilities, marked as *m*1 and *m*2. The scientists who have published at least one highly cited paper or at least one hot paper are characterized by their gender. The association of individuals with institution and with gender (when necessary) was based upon the POLON system, which is a central database of Polish scientists, and on the Web pages of their institutions.

## Results and discussion

The scientists from Lublin published about 1300 papers indexed in WoS^®^ in 2007 and about 2400 in 2016, and the number of papers increased linearly over the above period. Thus the number of papers under analysis was about 18,000 in highly cited, and about 4700 in hot papers. Out of these papers, 52 were highly cited, and 4 were hot on July 3, and 54 were highly cited, and 2 were hot on July 17. These numbers are substantially lower than 1% of 18,000, and lower than 1‰ of 4700, which are the expected numbers in a random set of the same size of papers from the database. This result is not surprising, because the scientific institutions of Lublin do not belong to the top universities of the world, and Pareto-type distribution of highly cited papers among the scientific institutions is expected. The number of hot papers from Lublin dropped by a factor of 2 in 2 weeks. This result is also in accordance with the expectations, namely the oldest papers in the set of 4700 have the highest chance of being hot (because they had sufficient time to collect many citations), but they are also sooner removed from the set (of papers which are less than 2 years old).

The results shown here demonstrate an extreme instability in time of any scientific indicator based upon hot papers. However such an indicator can still be used in assessment of top universities in the world, which produce hot papers by dozens (e.g., Harvard had 232 hot papers as of July 17, 2017) or of top journals (e.g., Nature had 132 hot papers as of July 17, 2017). As we focus on papers from Lublin in this paper, from now on we will discuss mainly highly cited papers, which are more stable in time, and will mention hot papers only occasionally. The highly cited papers from Lublin are analyzed one by one, and by author (all highly cited papers of one author considered as a group).

### Characterization of highly cited papers

The highly cited papers from Lublin are sorted by publication year in Table 1.

**Table 1:** Highly cited papers from Lublin sorted by publication year

Year	Highly cited 3.07	Highly cited 17.07
2007	7	7
2008	3	2
2009	4	4
2010	5	5
2011	2	2
2012	6	7
2013	5	5
2014	3	3
2015	9	8
2016	8	10
2017		1

The distribution by year shown in Table 1 does not reflect the linear increase in the aforementioned number of published papers from Lublin, and the numbers of highly cited papers vary by a factor of 5 between the best (2016) and the worst year (2011). This result is very different from the aforementioned linear increase of the count of highly cited papers from Malaysia with the publication year.

The highly cited papers from Lublin are sorted by research area in Table 2. Only the top research areas are indicated. The numbers attributed to certain research areas are not additive, that is, the same paper can be assigned to multiple research areas.

**Table 2:** Highly cited papers from Lublin sorted by research area

Discipline	Highly cited 3.07	Highly cited 17.07
Oncology	13	14
Chemistry	8	8
General internal medicine	7	7
Engineering	5	5
Environmental sciences ecology	3	3
Food science technology	3	3
Science technology other topics	3	3
Urology nephrology	3	4

The other research areas received 2 or less of highly cited papers from Lublin each. Table 2 indicates that in spite of the expected equalization of scientists representing various research areas, 80% of highly cited papers represent the top 8 research areas. The distribution of highly cited papers between research areas presented in Table 2 is very different from the aforementioned distributions in Malaysia, and in Russia.

The highly cited papers from Lublin are sorted by journal impact factor (for 2016) in Table 3.

**Table 3:** Highly cited papers from Lublin sorted by journal impact factor (for 2016)

IF	Highly cited 3.07	Highly cited 17.07
< 1	2	2
1–2	2	2
2–4	5	8
4–8	13	12
8–16	4	4
16–32	12	12
> 32	14	14

Interestingly enough, a substantial fraction of highly cited papers appeared in relatively low-IF-journals. Thus an assessment of publications discussed in this paper is weakly correlated with the assessment of publications by the journal IF.

The highly cited papers from Lublin are sorted by the number of authors in Table 4.

**Table 4:** Highly cited papers from Lublin sorted by the number of authors

Authors	Highly cited 3.07	Highly cited 17.07
1	2	2
2	5	6
3	5	5
4	4	5
5	3	2
6	1	1
7	2	2
8	2	2
9	0	0
10 +	28	29

Most highly-cited papers from Lublin have 10 authors or more. This problem is faced in most citation-based indicators, namely many successful papers are co-authored by numerous individuals, and the actual contribution of certain individual may be very little. The co-authors of certain paper can be from the same scientific institution, but they can very well be from different institution, different city or even from different country. The fraction of papers authored by 10 or more is much higher than the aforementioned analogous fraction of highly cited papers from Malaysia. On the other hand mega-authorship (1000 + authors) which contributed in 6% to highly cited papers from Malaysia was completely absent in highly cited papers from Lublin (the highest number of authors was 60).

Let us introduce a fractional authorship from Lublin (analogous definitions apply to different cities, countries, institutions, etc.) as the number of co-authors from Lublin divided by the total number of co-authors. The present definition is different from that by Pislyakov and Shukshina (2014) who performed similar analysis but with fractional affiliation (from Russia). The highly cited papers from Lublin are sorted by the fractional authorship from Lublin in Table 5.

**Table 5:** Highly cited papers from Lublin sorted by the fractional authorship from Lublin

Fractional authorship from Lublin	Highly cited 3.07	Highly cited 17.07
1	12	13
0.5–0.99	4	4
0.2–0.49	5	5
0.1–0.19	5	5
< 0.1	26	27

Most highly-cited papers from Lublin have very low (< 0.2) fractional authorship from Lublin. Many highly-cited papers from Lublin have only authors from Lublin. Relatively few highly-cited papers from Lublin have relatively high (0.2–0.99) contribution of authors from Lublin, but they also have co-authors from other cities/countries. Table 5 indicates that the aforementioned numbers (52 and 54) of highly cited papers substantially overrate the actual contribution of Lublin to the highly cited papers. Counting the highly cited papers fractionally (number of co-authors from Lublin divided by the total number of co-authors), the corresponding numbers of highly cited papers from Lublin are 18 and 19, respectively.

### Characterization of scientists by count of highly cited papers

Given that most highly cited papers are multi-author papers, the assessment of individuals and or of institutions based on highly cited papers is considered in two versions. In the first version each co-author takes the full credit for the entire paper. In the second version each co-author takes the only a reciprocal number of authors of a paper as a credit for the paper. Equal credit for each co-author is one of possible solutions, and other solutions (for example the first author receives more credit than the other authors) have been also considered in the literature, but detailed discussion of this problem is outside the scope of the present paper.

Achievements of authors of highly cited papers from Lublin are summarized in Table S1 in the Online Supporting Material. On top of the authors of highly cited papers, a few top scientists from Lublin (still active), who are not authors of highly cited papers are included for comparison. The scientists are marked *s*1, *s*2, … and they are sorted by the total number of citations.

Table S1 shows that most authors of highly cited papers have rather moderate achievements in terms of their scientific degrees, their total numbers of citations and of their Hirsch index. Holders of M.Sci. and Ph.D. (without habilitation) are commonplace. This should be emphasized that the total number of holders of a professor degree associated with scientific institutions of Lublin exceeds 500, and total number of holders of habilitation in Lublin (excluding professors) exceeds 1200. These numbers are higher by an order of magnitude than the number of highly cited papers from Lublin. Most authors of highly cited papers have fewer than 1000 citations, which is very little in comparison with the highest numbers indicated in the top part of Table S1. Table S1 shows a high degree of rank–rank correlation between the number of citations, *h*-index, and scientific degree, which are over 0.9 (citations vs. *h*-index), and about 0.8 (citations vs. rank, and *h*-index vs. rank), respectively. For example all scientists with > 2000 citations, and all scientist with *h* > 25 are professors. Table S1 shows a high degree of gender disparity in the number of citations, and in the *h*-index. For example all scientists but one with *h* > 25 are male.

The assessment of scientists by the number of highly cited papers as studied on July 3 is presented in Table S2 in the Online Supporting Material. The scientists are ordered by the fractional number of highly cited papers (each paper is taken with the weight of the reciprocal number of authors). There are 21 professors, 20 Drs.Sci., 17 Ph.Ds, and 6 Ms.Sci. among the authors of highly cited papers, and 3 Drs.Sci., 3 Ph.Ds, and 1 M.Sci. among the authors of hot papers (no single professor!). 29 scientists represent *u*1, 20 scientists represent *u*2, 3 scientists represent *u*3, 5 scientists represent *u*4, 1 scientist represents *u*5, 3 scientists represent *i*1, 2 scientists represent *m*1, and 1 scientist represents *m*2. These figures are nearly proportional to the overall scientific strengths of particular institutions as expressed by their total numbers of citations, except the achievements of *u*1 are slightly overrated.

Most scientists listed in Table S2 authored only one highly cited paper each, with the exception of one (female!) scientist who authored 9, one scientist who authored 3, and 6 scientists who authored 2 highly cited papers each. The achievements in terms of highly cited papers show little correlation with the achievements of the same scientist in terms of their scientific degrees, their total numbers of citations and of their Hirsch index as shown in Table 6. This should be emphasized that on top of the scientists shown in Table S2, there are numerous scientists in Lublin who have high scientific degrees, high numbers of citations, and high Hirsch indices, but zero highly cited papers (a few examples are given in Table S1).

**Table 6:** Rank–rank correlation coefficients between the number of highly cited papers on July 3 (total and fractional), scientific degree, total number of citations, and Hirsch index

	Highly cited total	Highly cited fractional	Citations	*h*	Academic degree
Highly cited total	1	0.25	0.29	0.23	0.17
Highly cited fractional	0.25	1	0.06	0.17	− 0.09
Citations	0.29	0.06	1	0.92	0.78
*h*	0.23	0.17	0.92	1	0.78
Academic degree	0.17	− 0.09	0.78	0.78	1

Table 6 shows that a high scientific degree, high total number of citations, and high Hirsch index do not imply that certain scientist is capable of producing highly cited paper(s).

The assessment of scientists by the number of highly cited papers as studied on July 17 is presented in Table S3 in the Online Supporting Material. The scientists are ordered by the fractional number of highly cited papers. There are 23 professors, 19 Drs. Sci., 18 Ph.Ds, and 5 Ms. Sci. among the authors of highly cited papers, and 2 Ph.Ds among the authors of hot papers (no single professor or Dr. Sci.!). 32 scientists represent *u*1, 16 scientists represent *u*2, 4 scientists represent *u*3, 5 scientists represent *u*4, 1 scientist represents *u*5, 3 scientists represent *i*1, 2 scientists represent *m*1, and 2 scientists represent *m*2. The achievements of *u*1 in terms of highly cited papers are substantially overrated with respect to other institutions as compared with the ranking based on overall scientific strength of this university as expressed by its total number of citations.

Most scientists listed in Table S3 authored only one highly cited paper each, with the exception of one scientist who authored 9 papers, two scientists who authored 3 papers each, and 5 scientists who authored 2 highly cited papers each. The ranks of particular scientists in Tables S2 and S3 are relatively consistent with very few exceptions. S15 advanced from rank 42 on July 3 to rank 5 on July 17 in fractional count of highly cited papers, and s34 who did not have any highly cited paper on July 3 advanced to rank 12 on July 17 in fractional count of highly cited papers. These few exceptions show that the position of particular scientists in the ranking can change substantially just in 2 weeks.

The achievements in terms of highly cited papers show little correlation with the achievements of the same scientist in terms of their scientific degrees, their total numbers of citations and of their Hirsch index as shown in Table 7.

**Table 7:** Rank–rank correlation coefficients between the number of highly cited papers on July 17 (total and fractional), scientific degree, total number of citations, and Hirsch index

	Highly cited total	Highly cited fractional	Citations	*h*	Academic degree
Highly cited total	1	0.34	0.27	0.21	0.16
Highly cited fractional	0.34	1	0.08	0.18	− 0.07
Citations	0.27	0.08	1	0.79	0.79
*h*	0.21	0.18	0.79	1	0.92
Academic degree	0.16	− 0.07	0.79	0.92	1

Table 7 shows that a high scientific degree, high total number of citations, and high Hirsch index do not imply that certain scientist is capable of producing highly cited paper(s). The male to female ratios in Tables S2 and S3 listing the authors of highly cited papers from Lublin are about 2:1. This indicates substantial domination of male authors. However this domination is still less substantial than in other aspects of academic career in Lublin, e.g., none of the five major universities of Lublin had ever a female rector. Also Table S1 shows substantial male domination in highly cited and in high-*h* scientists.

## Conclusions

The count of highly cited papers (total or fractional) can be used in assessment of scientific performance of individuals. The rankings based upon highly cited papers are extremely unstable in time, and the positions of individuals can substantially vary from one update of the list highly cited papers by WoS^®^ to another. The rankings of scientists based upon highly cited papers are not correlated with the rankings based upon the academic degrees, numbers of citations or on the Hirsch-indices. The rankings of scientists based upon highly cited papers reduce the inherent advantage of old scientists and of scientists working in more-popular disciplines, which is the case with total number of publications, total number of citations and with the Hirsch-index. The rankings of scientists based upon highly cited papers suffer several shortages, which are common for most citation-based indices, e.g., they can be manipulated by excessive self-citations.

## Electronic supplementary material

Below is the link to the electronic supplementary material.

Supplementary material 1 (DOC 388 kb)

## References

[CR1] Bornmann L, Ruediger H, Sven E (2011). A multilevel meta-analysis of studies reporting correlations between the *h* index and 37 different *h* index variants. Journal of Informetrics.

[CR2] Docampo D, Cram L (2017). Academic performance and institutional resources: A cross-country analysis of research universities. Scientometrics.

[CR3] Garfield E (1955). Citation indexes for science—new dimension in documentation through association of ideas. Science.

[CR4] Garfield E (2006). The history and meaning of the journal impact factor. JAMA-Journal of the American Medical Association.

[CR5] Hirsch JE (2005). An index to quantify an individual’s scientific research output. Proceedings of the National academy of Sciences of the United States of America.

[CR6] Kosmulski M (2011). Successful papers: A new idea in evaluation of scientific output. Journal of Informetrics.

[CR7] Miryairi N, Chang HW (2012). Bibliometric characteristics of highly cited papers from Taiwan, 2000–2009. Scientometrics.

[CR8] Noorhidawati A, Yanti Idata Aspura MK, Zahila MN, Abrizah A (2017). Characteristics of Malaysian highly cited papers. Malaysian Journal of Library & Information Science.

[CR9] Pislyakov V, Shukshina E (2014). Measuring excellence in Russia: Highly cited papers, leading institutions, patterns of national and international collaboration. Journal of the Association for Information Science and Technology.

[CR10] Plomp R (1990). The significance of the number of highly cited papers as an indicator of scientific prolificacy. Scientometrics.

[CR11] Schreiber M (2010). Twenty Hirsch index variants and other indicators giving more or less preference to highly cited papers. Annalen der Physik.

