# Supplementary material for: Are you in top 1% (1‰)?
Source: Scientometrics. 2017 Sep 23;114(2):557–65. doi: 10.1007/s11192-017-2526-4 (PMC5807554; doi:10.1007/s11192-017-2526-4)
Supplement: Supplementary file 1 — Supplementary material 1 (DOC 388 kb) [file 11192_2017_2526_MOESM1_ESM.doc]

Are you in top 1% (1‰)?*

Marek Kosmulski

Lublin University of Technology, Lublin, Poland

Table S1. Authors of highly cited papers from Lublin

| name | sex | institution | Scientific degree | h | citations | Author of highly cited paper(s)? |
| --- | --- | --- | --- | --- | --- | --- |
| s01 | m | m2 | 3 | 49 | 8595 | yes |
| s02 | m | u1 | 3 | 46 | 8594 | no |
| s03 | f | u1 | 3 | 36 | 8282 | yes |
| s04 | m | u2 | 3 | 19 | 7459 | no |
| s05 | m | u1 | 3 | 38 | 6658 | no |
| s06 | m | u2 | 3 | 31 | 4899 | no |
| s07 | m | u2 | 3 | 34 | 4451 | no |
| s08 | m | u4 | 3 | 30 | 3766 | yes |
| s09 | m | u2 | 3 | 33 | 3764 | yes |
| s10 | f | m1 | 3 | 9 | 3680 | yes |
| s11 | m | u2 | 3 | 31 | 3529 | yes |
| s12 | m | u1 | 3 | 28 | 3436 | yes |
| s13 | m | m2 | 3 | 28 | 2904 | yes |
| s14 | m | u2 | 3 | 22 | 2754 | yes |
| s15 | m | u1 | 3 | 20 | 1988 | yes |
| s16 | f | u1 | 3 | 17 | 1893 | yes |
| s17 | m | u2 | 2 | 23 | 1755 | yes |
| s18 | m | u1 | 3 | 16 | 1754 | yes |
| s19 | m | u1 | 3 | 18 | 1515 | yes |
| s20 | m | u1 | 1 | 11 | 1504 | yes |
| s21 | f | u1 | 3 | 22 | 1439 | yes |
| s22 | m | u1 | 3 | 18 | 1416 | yes |
| s23 | m | u2 | 2 | 21 | 1361 | yes |
| s24 | f | u1 | 3 | 16 | 1182 | yes |
| s25 | m | u1 | 3 | 15 | 1175 | yes |
| s26 | m | u1 | 3 | 17 | 1067 | yes |
| s27 | m | u2 | 2 | 15 | 1047 | yes |
| s28 | m | u1 | 3 | 12 | 996 | yes |
| s29 | m | u1 | 3 | 13 | 920 | yes |
| s30 | m | u1 | 1 | 13 | 897 | yes |
| s31 | f | u3 | 2 | 18 | 862 | yes |
| s32 | m | u1 | 2 | 16 | 805 | yes |
| s33 | m | u2 | 3 | 16 | 727 | yes |
| s34 | m | u1 | 2 | 14 | 721 | yes |
| s35 | f | u2 | 2 | 14 | 699 | yes |
| s36 | m | u3 | 2 | 16 | 669 | yes |
| s37 | m | i1 | 3 | 14 | 648 | yes |
| s38 | f | u2 | 2 | 13 | 537 | yes |
| s39 | m | u4 | 2 | 14 | 534 | yes |
| s40 | m | u2 | 2 | 11 | 521 | yes |
| s41 | f | u2 | 2 | 13 | 507 | yes |
| s42 | f | m1 | 1 | 5 | 460 | yes |
| s43 | f | u2 | 2 | 11 | 454 | yes |
| s44 | m | u1 | 3 | 8 | 447 | yes |
| s45 | m | u1 | 2 | 10 | 440 | yes |
| s46 | f | u1 | 1 | 5 | 428 | yes |
| s47 | m | u4 | 3 | 11 | 414 | yes |
| s48 | m | u2 | 1 | 8 | 411 | yes |
| s49 | m | u1 | 1 | 9 | 405 | yes |
| s50 | f | u1 | 1 | 8 | 401 | yes |
| s51 | f | u1 | 1 | 8 | 373 | yes |
| s52 | f | u1 | 2 | 5 | 358 | yes |
| s53 | m | i1 | 2 | 7 | 346 | yes |
| s54 | m | u1 | 1 | 4 | 282 | yes |
| s55 | f | u2 | 1 | 9 | 265 | yes |
| s56 | m | u5 | 2 | 10 | 259 | yes |
| s57 | m | u1 | 2 | 8 | 246 | yes |
| s58 | m | u1 | 1 | 8 | 219 | yes |
| s59 | m | i1 | 2 | 8 | 211 | yes |
| s60 | f | u4 | 1 | 8 | 210 | yes |
| s61 | m | u1 | 2 | 8 | 197 | yes |
| s62 | m | u2 | 1 | 5 | 150 | yes |
| s63 | f | u2 | 2 | 6 | 132 | yes |
| s64 | f | u1 | 1 | 7 | 120 | yes |
| s65 | m | u3 | 0 | 6 | 89 | yes |
| s66 | m | u3 | 1 | 5 | 88 | yes |
| s67 | f | u4 | 1 | 4 | 82 | yes |
| s68 | m | u1 | 0 | 5 | 74 | yes |
| s69 | f | u2 | 1 | 4 | 64 | yes |
| s70 | f | u1 | 1 | 2 | 55 | yes |
| s71 | f | u2 | 0 | 4 | 43 | yes |
| s72 | f | u2 | 0 | 1 | 37 | yes |
| s73 | f | u1 | 0 | 2 | 25 | yes |
| s74 | m | u2 | 0 | 1 | 22 | yes |
| s75 | f | u1 | 1 | 3 | 14 | yes |
| s76 | f | u1 | 0 | 1 | 1 | yes |

Table S2 The assessment of scientists by the number of highly cited papers as studied on July 3

| name | # fractional | # total | Rank fractional | Rank total |
| --- | --- | --- | --- | --- |
| s08 | 1 | 1 | 1.5 | 37 |
| s23 | 1 | 1 | 1.5 | 37 |
| s32 | 0.7 | 2 | 3.5 | 5.5 |
| s68 | 0.7 | 2 | 3.5 | 5.5 |
| s35 | 0.666667 | 2 | 5.5 | 5.5 |
| s17 | 0.666667 | 2 | 5.5 | 5.5 |
| s03 | 0.56557 | 9 | 7 | 1 |
| s47 | 0.5 | 1 | 10.5 | 37 |
| s12 | 0.5 | 1 | 10.5 | 37 |
| s21 | 0.5 | 1 | 10.5 | 37 |
| s46 | 0.5 | 1 | 10.5 | 37 |
| s51 | 0.5 | 1 | 10.5 | 37 |
| s72 | 0.5 | 1 | 10.5 | 37 |
| s19 | 0.333333 | 1 | 19 | 37 |
| s28 | 0.333333 | 1 | 19 | 37 |
| s30 | 0.333333 | 1 | 19 | 37 |
| s50 | 0.333333 | 1 | 19 | 37 |
| s11 | 0.333333 | 1 | 19 | 37 |
| s55 | 0.333333 | 1 | 19 | 37 |
| s62 | 0.333333 | 1 | 19 | 37 |
| s69 | 0.333333 | 1 | 19 | 37 |
| s31 | 0.333333 | 1 | 19 | 37 |
| s36 | 0.333333 | 1 | 19 | 37 |
| s65 | 0.333333 | 1 | 19 | 37 |
| s37 | 0.25 | 1 | 28.5 | 37 |
| s39 | 0.25 | 1 | 28.5 | 37 |
| s60 | 0.25 | 1 | 28.5 | 37 |
| s67 | 0.25 | 1 | 28.5 | 37 |
| s70 | 0.25 | 1 | 28.5 | 37 |
| s09 | 0.25 | 1 | 28.5 | 37 |
| s27 | 0.25 | 1 | 28.5 | 37 |
| s48 | 0.25 | 1 | 28.5 | 37 |
| s75 | 0.2 | 1 | 37 | 37 |
| s41 | 0.2 | 1 | 37 | 37 |
| s63 | 0.2 | 1 | 37 | 37 |
| s71 | 0.2 | 1 | 37 | 37 |
| s74 | 0.2 | 1 | 37 | 37 |
| s56 | 0.2 | 1 | 37 | 37 |
| s57 | 0.2 | 1 | 37 | 37 |
| s61 | 0.2 | 1 | 37 | 37 |
| s76 | 0.2 | 1 | 37 | 37 |
| s15 | 0.183333 | 2 | 42 | 5.5 |
| s14 | 0.166667 | 1 | 43 | 37 |
| s10 | 0.155263 | 3 | 44 | 2 |
| s26 | 0.142857 | 1 | 45.5 | 37 |
| s40 | 0.142857 | 1 | 45.5 | 37 |
| s29 | 0.125 | 1 | 47.5 | 37 |
| s52 | 0.125 | 1 | 47.5 | 37 |
| s25 | 0.095556 | 2 | 49 | 5.5 |
| s38 | 0.090909 | 1 | 50.5 | 37 |
| s43 | 0.090909 | 1 | 50.5 | 37 |
| s01 | 0.083333 | 1 | 52 | 37 |
| s18 | 0.0625 | 1 | 53 | 37 |
| s54 | 0.055556 | 1 | 54.5 | 37 |
| s45 | 0.055556 | 1 | 54.5 | 37 |
| s53 | 0.052632 | 1 | 56 | 37 |
| s42 | 0.05 | 1 | 57 | 37 |
| s16 | 0.047619 | 1 | 58 | 37 |
| s33 | 0.041667 | 1 | 59 | 37 |
| s58 | 0.038462 | 1 | 60 | 37 |
| s49 | 0.035714 | 1 | 61 | 37 |
| s44 | 0.025 | 1 | 62 | 37 |
| s59 | 0.02 | 1 | 63 | 37 |
| s20 | 0.016667 | 1 | 64 | 37 |

Table S3 The assessment of scientists by the number of highly cited papers as studied on July 17

| name | # fractional | # total | Rank fractional | Rank total |
| --- | --- | --- | --- | --- |
| s23 | 1 | 1 | 1.5 | 37 |
| s08 | 1 | 1 | 1.5 | 37 |
| s68 | 0.7 | 2 | 3.5 | 6 |
| s32 | 0.7 | 2 | 3.5 | 6 |
| s15 | 0.683333 | 3 | 5 | 2.5 |
| s35 | 0.666667 | 2 | 6.5 | 6 |
| s17 | 0.666667 | 2 | 6.5 | 6 |
| s03 | 0.56557 | 9 | 8 | 1 |
| s72 | 0.5 | 1 | 12 | 37 |
| s51 | 0.5 | 1 | 12 | 37 |
| s47 | 0.5 | 1 | 12 | 37 |
| s46 | 0.5 | 1 | 12 | 37 |
| s34 | 0.5 | 1 | 12 | 37 |
| s21 | 0.5 | 1 | 12 | 37 |
| s12 | 0.5 | 1 | 12 | 37 |
| s69 | 0.333333 | 1 | 21 | 37 |
| s65 | 0.333333 | 1 | 21 | 37 |
| s62 | 0.333333 | 1 | 21 | 37 |
| s55 | 0.333333 | 1 | 21 | 37 |
| s50 | 0.333333 | 1 | 21 | 37 |
| s36 | 0.333333 | 1 | 21 | 37 |
| s31 | 0.333333 | 1 | 21 | 37 |
| s30 | 0.333333 | 1 | 21 | 37 |
| s28 | 0.333333 | 1 | 21 | 37 |
| s19 | 0.333333 | 1 | 21 | 37 |
| s11 | 0.333333 | 1 | 21 | 37 |
| s73 | 0.25 | 1 | 32.5 | 37 |
| s70 | 0.25 | 1 | 32.5 | 37 |
| s67 | 0.25 | 1 | 32.5 | 37 |
| s66 | 0.25 | 1 | 32.5 | 37 |
| s64 | 0.25 | 1 | 32.5 | 37 |
| s60 | 0.25 | 1 | 32.5 | 37 |
| s48 | 0.25 | 1 | 32.5 | 37 |
| s39 | 0.25 | 1 | 32.5 | 37 |
| s37 | 0.25 | 1 | 32.5 | 37 |
| s27 | 0.25 | 1 | 32.5 | 37 |
| s24 | 0.25 | 1 | 32.5 | 37 |
| s09 | 0.25 | 1 | 32.5 | 37 |
| s76 | 0.2 | 1 | 40.5 | 37 |
| s61 | 0.2 | 1 | 40.5 | 37 |
| s57 | 0.2 | 1 | 40.5 | 37 |
| s56 | 0.2 | 1 | 40.5 | 37 |
| s14 | 0.166667 | 1 | 43 | 37 |
| s10 | 0.155263 | 3 | 44 | 2.5 |
| s40 | 0.142857 | 1 | 45.5 | 37 |
| s26 | 0.142857 | 1 | 45.5 | 37 |
| s52 | 0.125 | 1 | 47.5 | 37 |
| s29 | 0.125 | 1 | 47.5 | 37 |
| s13 | 0.1 | 1 | 49 | 37 |
| s25 | 0.095556 | 2 | 50 | 6 |
| s43 | 0.090909 | 1 | 51.5 | 37 |
| s38 | 0.090909 | 1 | 51.5 | 37 |
| s01 | 0.083333 | 1 | 53 | 37 |
| s18 | 0.0625 | 1 | 54 | 37 |
| s54 | 0.055556 | 1 | 55.5 | 37 |
| s45 | 0.055556 | 1 | 55.5 | 37 |
| s53 | 0.052632 | 1 | 57 | 37 |
| s42 | 0.05 | 1 | 58 | 37 |
| s33 | 0.041667 | 1 | 59.5 | 37 |
| s22 | 0.041667 | 1 | 59.5 | 37 |
| s58 | 0.038462 | 1 | 61 | 37 |
| s49 | 0.035714 | 1 | 62 | 37 |
| s44 | 0.025 | 1 | 63 | 37 |
| s59 | 0.02 | 1 | 64 | 37 |
| s20 | 0.016667 | 1 | 65 | 37 |

Note added in proof.

In course of production of the present article, the lists of highly cited and of hot papers were updated again. A few results related to the recent update (database accessed on September 14, 2017 are reported below.

The number of highly cited papers from Lublin increased to 58, and the number of hot papers dropped to 1. The only hot paper on September 14 was also hot on July 3 and on July 17. Out of 58 highly cited papers, 50 were also highly cited on July 3 and on July 17, 1 was also highly cited on July 3 but not on July 17, 3 were also highly cited on July 17 but not on July 3, and 4 papers were new on the list (absent on the list of highly cited papers on July 3 and on July 17). Automatic search produced 59 hits, but one result was due to a spelling error, and actually the author was from another city in Poland, which is Lubin. The name of the city was correct in the original article, but is was incorrect in WoS®. Three out of 4 papers, which appeared on the list of highly cited papers on September 14, but not on July 3 or on July 17 had co-authors, who are not included in Table S1. These scientists are introduced in Table S4.

Table S4. New authors of highly cited papers from Lublin on September 14

| name | sex | institution | Scientific degree | h | citations | Author of highly cited paper(s)? |
| --- | --- | --- | --- | --- | --- | --- |
| s77 | f | u2 | 2 | 8 | 321 | yes |
| s78 | f | u3 | 3 | 9 | 297 | yes |
| s79 | f | u3 | 1 | 4 | 138 | yes |
| s80 | f | u2 | 0 | 1 | 10 | yes |

The updated ranking of scientists from Lublin based upon the fractional counting of highly cited papers is presented in Table S5.

Table S5. The assessment of scientists by the number of highly cited papers as studied on September 14

| name | # fractional | # total | Rank fractional | Rank total |
| --- | --- | --- | --- | --- |
| s68 | 1.2 | 3 | 1 | 4 |
| s32 | 1.2 | 3 | 1 | 4 |
| s35 | 1 | 3 | 4.5 | 4 |
| s23 | 1 | 1 | 4.5 | 39 |
| s08 | 1 | 1 | 4.5 | 39 |
| s77 | 1 | 1 | 4.5 | 39 |
| s15 | 0.683333 | 3 | 7 | 4 |
| s17 | 0.666667 | 2 | 8 | 7.5 |
| s03 | 0.56557 | 9 | 9 | 1 |
| s51 | 0.5 | 1 | 14 | 39 |
| s21 | 0.5 | 1 | 14 | 39 |
| s72 | 0.5 | 1 | 14 | 39 |
| s34 | 0.5 | 1 | 14 | 39 |
| s46 | 0.5 | 1 | 14 | 39 |
| s12 | 0.5 | 1 | 14 | 39 |
| s47 | 0.5 | 1 | 14 | 39 |
| s78 | 0.5 | 1 | 14 | 39 |
| s79 | 0.5 | 1 | 14 | 39 |
| s50 | 0.333333 | 1 | 24.5 | 39 |
| s11 | 0.333333 | 1 | 24.5 | 39 |
| s69 | 0.333333 | 1 | 24.5 | 39 |
| s30 | 0.333333 | 1 | 24.5 | 39 |
| s28 | 0.333333 | 1 | 24.5 | 39 |
| s19 | 0.333333 | 1 | 24.5 | 39 |
| s65 | 0.333333 | 1 | 24.5 | 39 |
| s36 | 0.333333 | 1 | 24.5 | 39 |
| s31 | 0.333333 | 1 | 24.5 | 39 |
| s62 | 0.333333 | 1 | 24.5 | 39 |
| s55 | 0.333333 | 1 | 24.5 | 39 |
| s80 | 0.3333 | 1 | 24.5 | 39 |
| s73 | 0.25 | 1 | 36.5 | 39 |
| s66 | 0.25 | 1 | 36.5 | 39 |
| s64 | 0.25 | 1 | 36.5 | 39 |
| s24 | 0.25 | 1 | 36.5 | 39 |
| s67 | 0.25 | 1 | 36.5 | 39 |
| s60 | 0.25 | 1 | 36.5 | 39 |
| s39 | 0.25 | 1 | 36.5 | 39 |
| s37 | 0.25 | 1 | 36.5 | 39 |
| s70 | 0.25 | 1 | 36.5 | 39 |
| s27 | 0.25 | 1 | 36.5 | 39 |
| s48 | 0.25 | 1 | 36.5 | 39 |
| s09 | 0.25 | 1 | 36.5 | 39 |
| s76 | 0.2 | 1 | 44.5 | 39 |
| s61 | 0.2 | 1 | 44.5 | 39 |
| s57 | 0.2 | 1 | 44.5 | 39 |
| s56 | 0.2 | 1 | 44.5 | 39 |
| s14 | 0.166667 | 1 | 47 | 39 |
| s10 | 0.155263 | 3 | 48 | 4 |
| s26 | 0.142857 | 1 | 49.5 | 39 |
| s40 | 0.142857 | 1 | 49.5 | 39 |
| s52 | 0.125 | 1 | 51.5 | 39 |
| s29 | 0.125 | 1 | 51.5 | 39 |
| s25 | 0.095556 | 2 | 53 | 7.5 |
| s43 | 0.090909 | 1 | 54.5 | 39 |
| s38 | 0.090909 | 1 | 54.5 | 39 |
| s01 | 0.083333 | 1 | 56 | 39 |
| s18 | 0.0625 | 1 | 57 | 39 |
| s54 | 0.055556 | 1 | 58.5 | 39 |
| s45 | 0.055556 | 1 | 58.5 | 39 |
| s53 | 0.052632 | 1 | 60 | 39 |
| s42 | 0.05 | 1 | 61 | 39 |
| s16 | 0.047619 | 1 | 62 | 39 |
| s33 | 0.041667 | 1 | 63.5 | 39 |
| s22 | 0.041667 | 1 | 63.5 | 39 |
| s58 | 0.038462 | 1 | 65 | 39 |
| s49 | 0.035714 | 1 | 66 | 39 |
| s44 | 0.025 | 1 | 67 | 39 |
| s59 | 0.02 | 1 | 68 | 39 |
| s20 | 0.016667 | 1 | 69 | 39 |

The ranks of particular scientists in Table S4 are relatively consistent with their ranks in Tables S2 and S3 with a few exceptions. Namely, S77, S78, S79, and S80, who were absent in the previous two rankings, have ranks as high as 4.5, 14, 14, and 24.5, respectively in the current ranking.
